# Variceal Band Ligation in the Prevention of Variceal Bleeding: A Multicenter Trial

**DOI:** 10.4103/1319-3767.77238

**Published:** 2011

**Authors:** Asma Ouakaa-Kchaou, Jamel Kharrat, Khaoula Mir, Boussourra Houda, Nabil Abdelli, Salem Ajmi, Msaddek Azzouz, Hatem Ben Abdallah, Nabyl Ben Mami, Slim Bouzaidi, Sofiene Chouaib, Lamia Golli, Wissem Melki, Taoufik Najjar, Hammouda Saffar, Najet Belhadj, Abdeljabbar Ghorbel

**Affiliations:** Department of Gastroenterology, Habib Thameur Hospital, Tunis, Tunisia; 1 Department of Gastroenterology, Military Hospital, Tunis, Tunisia; 2 Department of Gastroenterology, Sahloul Hospital, Sousse, Tunisia; 3 Department of Gastroenterology, Maamouri Hospital, Nabeul, Tunisia; 4 Department of Gastroenterology, B, La Rabta Hospital, Tunis, Tunisia; 5 Department of Gastroenterology, Charles Nicolle Hospital, Tunis, Tunisia; 6 Department of Gastroenterology, Fattouma Bourguiba Hospital, Monastir, Tunisia; 7 Department of Gastroenterology, Mongi Slim Hospital, Tunis, Tunisia

**Keywords:** Endoscopic band ligation, esophageal varices, portal hypertension

## Abstract

**Background/Aim:**

Variceal bleeding is a life-threatening complication of portal hypertension with a high probability of recurrence. Treatment to prevent first bleeding or rebleeding is mandatory. The study has been aimed at investigating the effectiveness of endoscopic band ligation in preventing upper gastrointestinal bleeding in patients with portal hypertension and to establish the clinical outcome of patients.

**Patients and Methods:**

We analyzed in a multicenter trial, the efficacy and side effects of endoscopic band ligation for the primary and secondary prophylaxis of esophageal variceal bleeding. We assigned 603 patients with portal hypertension who were hospitalized to receive treatment with endoscopic ligation. Sessions of ligation were repeated every two to three weeks until the varices were eradicated. The primary end point was recurrent bleeding.

**Results:**

The median follow-up period was 32 months. A total of 126 patients had recurrent bleeding. All episodes were related to portal hypertension and 79 to recurrent variceal bleeding. There were major complications in 51 patients (30 had bleeding esophageal ulcers). Seventy-eight patients died, 26 deaths were related to variceal bleeding and 1 to bleeding esophageal ulcers.

**Conclusions:**

A great improvement in the prevention of variceal bleeding has emerged over the last years. However, further therapeutic options that combine higher efficacy, better tolerance and fewer side effects are needed.

Variceal bleeding is a life-threatening complication of portal hypertension with a high probability of recurrence.[1]

Patients surviving a first episode of variceal bleeding have a risk of over 60% of experiencing recurrent hemorrhage within two years from the index episode. As a consequence, treatment to prevent first bleeding is mandatory, as well; all patients surviving a variceal bleed must receive active treatments to prevent rebleeding.[1]

Endoscopic elastic band ligation (EBL) in eradicating esophageal varices has been shown to be an effective, safe, easy-to-do procedure with few untoward effects.[1–3]

Therefore, the present study has been aimed at investigating, in a multicenter trial, whether endoscopic band ligation is effective in preventing upper gastrointestinal bleeding in patients with portal hypertension and to establish the clinical outcome of patients.

## PATIENTS AND METHODS

### Selection of patients

This retrospective trial was conducted in seven hospitals in Tunisia. Consecutive patients with portal hypertension referred between January 1998 and December 2007 to any of the seven participating hospitals for endoscopic band ligation were considered.

Exclusion criteria were advanced hepatocellular carcinoma, a concomitant disease with reduced life expectancy, previous treatment to prevent bleeding with a portosystemic shunt or with EBL, bleeding from isolated gastric or ectopic varices.

### Baseline evaluation and general management

A full clinical history, physical examination, electrocardiogram, chest radiograph, laboratory tests and ultrasonography were performed. Propranolol was initiated. The dose was increased stepwise, every two–three days, up to the maximum tolerated dose or up to 160 mg/day.

The first elective session was carried out within a mean of 12 days from the indication. Then EBL sessions were scheduled at an average of every 14 days until variceal eradication (disappearance of varices or being too small to be sucked in the banding device). EBL sessions were initially performed using single-band device with or without overtube; then with multiband ligation devices (SpeedBand, Boston Scientific, or Six Shooter Multi-Band ligator, Cook Medical) when they became commercially available; application of the bands was started at the gastro-esophageal junction and progressed upward in a helical way for approximately 5–8 cm. Procedures were performed under local sedation with xylocain.

### Follow-up

After eradication, an endoscopic control was performed at one, three, six months and then yearly to monitor variceal recurrence, which was defined as the reappearance of varices, which could be ligated. Variceal recurrences were treated with repeat EBL. All patients underwent clinical reassessment every six months or more often if needed. Median follow-up was 32 months (range 2 – 212 months).

Untoward events considered to be related to treatment under study and requiring active therapy or prolonged hospitalization were recorded. Side effects were considered severe if the health or safety of the patient was endangered (i.e. pneumonia, sepsis, bacterial peritonitis or bleeding from esophageal ulcers). Minor symptoms or events, which were not considered worthy of treatment or investigation by the attending physician, were also recorded.

### End points

The main endpoint of the study was bleeding from any source (first bleeding for primary prophylaxis and recurrent bleeding for secondary prophylaxis). Secondary endpoints were the number of banding sessions, the time to eradication (calculated from the first banding session), the incidence of variceal recurrence, variceal rebleeding, complications and mortality (overall and mortality related to bleeding).

All patients were instructed to come to hospital whenever they experienced melena or hematemesis. If bleeding was confirmed, an emergency endoscopy was performed. The diagnosis of variceal bleeding was done when varices were actively bleeding or had stigmata of recent bleeding and/or if fresh blood was observed in the stomach and varices were the only potential source of bleeding. Bleeding was considered to be EBL-related when endoscopy disclosed bleeding from an ulcer secondary to previous ligation. The rebleeding episode was treated primarily by means of vasoactive drugs (somatostatin) and endoscopic treatment, preferably with EBL.

## RESULTS

Six hundred and three patients were included in the study. The indication of EBL was mainly a secondary prophylaxis (92%). The characteristics of the patients are shown in Table 1.

**Table 1:** Clinical characteristics of the patients included in the study

Characteristics	
Age (years)	55 (10 – 95)
Sex (male/female)	332 (55%) / 271 (45%)
Indication of band ligation	
Primary prophylaxis	49 (8%)
Secondary prophylaxis	554 (92%)
Etiology of portal hypertension	
Cirrhosis	91.5%
Portal cavernoma	6.8%
Nodular regenerative hyperplasia	1%
Congenital hepatic fibrosis	0.7%
Child Pugh class A/B/C (%)	22/56/22
Esophageal varices	
Grade II	186 (30.8%)
Grade III	417 (69.2%)
Active bleeding at endoscopy (spurting or oozing)	70
Recent bleeding stigmata	50
White nipple	28
Clot over a varix	22
Propranolol	454 (75.3%)

The cause of cirrhosis was hepatitis C (HCV) alone (34%), hepatitis B (HBV) alone (31%), cryptogenic (22.6%), combined HCV and HBV (1.28%), combined HBV and hepatitis D (0.37%), alcohol (3.8%), autoimmune hepatitis (3%), primary biliary cirrhosis (2.5%) and non-alcoholic fatty liver (1.45%).

Each varix was ligated at least once and up to seven bands were placed (mean four bands). Variceal eradication was achieved in 492 of the 603 patients (81.6%) with a mean of 3.5 EBL sessions (range 1–12 sessions) and after a median of nine weeks (range 2–32 weeks). The results of EBL according to the indication are resumed in Table 2. Concerning patient having active bleeding at endoscopy, the success rate of hemostasis achieved by ligation was 97%. Variceal eradication was not achieved in 111 patients (because of recurrent bleeding, death, lost to follow-up, or despite multiple EBL sessions). In 130 of the 492 patients achieving eradication (26%), varices reappeared after a median of 35 weeks after eradication. Recurrent varices were eradicated by one to three sessions of EBL (mean two sessions and seven bands) in 110 cases (84.6%).

**Table 2:** Results of endoscopic band ligation according to the indication

	Primary prophylaxis	Secondary prophylaxis
Number of patients	49	554
Interval between sessions	2 – 3 weeks	2 – 3 weeks
Number of sessions to eradication (mean)	3	3.5
Number of bands (mean)	12	14
Time to eradication (weeks)	8.5	9.5

At the time of the index endoscopy, 343 patients had portal hypertensive gastropathie. At the last endoscopy performed during follow-up, 400 patients had portal hypertensive gastropathie, among them two had a worsening of the pre-existent lesions, with severe features. Concerning gastric varices, 35 patients had worsened pre-existent varices and 51 developed secondary varices after EBL.

Propranolol was prescribed in association with EBL in 454 patients as a secondary prophylaxis. The dosage was variable according to the patient (until the heart rate had fallen by 25%).

### Bleeding after EBL

One hundred and twenty six patients bled during follow-up [Table 3]. In 30 patients bleeding was secondary to esophageal ulcers related to endoscopic treatment, in 15 to gastric varices and in 2 to portal hypertensive gastropathie. As a consequence, only 79 patients bled with confirmed esophageal variceal bleeding. The first variceal bleeding rate after EBL was 10.2% and the rebleeding rate was 13.3%. This variceal bleeding occurred less likely in patients on beta-blockers (15% vs. 18% in patients without beta-blockers) but the difference did not reach statistical significance.

**Table 3:** Episodes of recurrent bleeding

	*N*
Patients with recurrent bleeding	126
Total number of episodes	126
Site of recurrent bleeding (N)	
Esophageal varices	79
Esophageal ulcer	30
Portal hypertensive gastropathie	2
Gastric varices	15

### Survival

Overall, 78 patients died (13%). Only 27 deaths were bleeding-related (esophageal varices = 14 cases, gastric varices = 12 cases and esophageal ulcers = 1 case); the other causes of death were spontaneous ascites infection (20 cases), hepatorenal syndrome (six cases), liver failure (nine cases), hepatocellular carcinoma (10 cases) and non-liver related cause (six cases).

### Adverse effects

Overall, the number of patients with adverse events was 86. Major side effects occurred in 51 patients, which presented with esophageal ulcers. There was no case of aspiration pneumonia, bacterial peritonitis, empyema or sepsis.

Minor side effects were post-procedural chest pain (21 cases), fever (four cases), transient dysphagia (nine cases) and overtube’s migration (one case).

## DISCUSSION

The indications for EBL of esophageal varices include control of acute variceal bleeding, primary prophylaxis to prevent the first episode of variceal bleeding in high-risk patients, and secondary prophylaxis to prevent rebleeding following an initial episode of acute variceal bleeding.[2,3]

Hemostatic treatment is essential in acute esophageal variceal bleeding, a medical emergency associated with relevant morbidity and mortality.[4] By combining both therapies, the local hemostatic effect induced by endoscopic treatment on the varices is added to the portal hypotensive effect achieved with drugs. This combination is the therapy of choice currently recommended for acute variceal. The use of variceal ligation instead of sclerotherapy as emergency endoscopic therapy for the treatment of acute variceal bleeding significantly improves the efficacy and safety.[4] EBL controlled bleeding in 97% of our patients whereas therapeutic success was 90% in the study of Villanueva *et al*.[4]

Non-selective beta-blockers are the recommended first line therapy for the primary prophylaxis of variceal hemorrhage in patients with varices at high risk for bleeding. Beta-blockers reduce the two-year incidence of first bleeding in these patients by 40%.[5] However, beta-blockers are not suitable for all patients: contraindications may be present in 5–20% of potential candidates, and 9–33% may develop side effects that lead to discontinuation of the treatment in 3–27% of cases.[5] The optimal strategy to manage these patients is to treat with EBL, which achieve protection from variceal bleeding comparable to that of good responders to beta-blockers.[1,5,6] In this indication, the first bleeding rate was 10.2% comparable with that of other studies (8.9% in the study of Dell’Era *et al*.).[5]

After an episode of acute esophageal variceal bleeding, patients are at high risk for recurrent bleeding and death. Thus, therapy to prevent recurrent bleeding is essential.[1,7,8]

Endoscopic sclerotherapy is of proven benefit in such cases. However, it is associated with a rate of recurrent bleeding of up to 50% and with local and systemic complications such as fever, pain, pulmonary infections, and esophageal ulceration, stricture, and perforation. Some of these complications may be fatal. Endoscopic variceal ligation is a purely mechanical method of obliterating varices that was introduced to preclude the undesirable effects of sclerotherapy. Several studies have shown that, as compared with sclerotherapy, variceal ligation is safer, requires fewer sessions to obliterate varices, significantly reduces the rate of recurrent bleeding, and improves the probability of survival.[1,2,8]

Accordingly, endoscopic ligation is currently the preferred endoscopic treatment for preventing recurrent variceal bleeding. The efficacy of variceal ligation, as found in our study, is consistent with the higher ranges previously reported in randomized trials of this treatment. A relatively wide variation in rates of recurrent bleeding has been observed with ligation (10 to 50%, 13.3% in our study.[1,9,10] This variation may be due, at least in part, to technical differences among studies, such as variations in the interval between sessions or in the number of bands placed during each session. Moreover, in multi-site band ligation-treated patients, eradication of varices seems to be achieved with fewer sessions of treatment than in those treated with conventional band ligation.[11] Whether these or other technical differences can affect the outcome has not been adequately investigated. Other possible confounding factors, such as the time since the initial bleeding episode, alcohol use or non-use, and the treatment used to stop the bleeding, may also affect the results of treatment. Among different trials, there may be differences in the randomization process or in the characteristics of the population treated, such as the cause or the severity of hypertension, or in the definition of end points such as recurrent bleeding.[12–15] Our study had few exclusion criteria; a high proportion of the patients had advanced liver disease. A combination of non-selective beta-blockers and EBL may be the best alternative.[1,16,17]

It has also been suggested that, as with sclerotherapy, variceal ligation may worsen the severity of portal hypertensive gastropathie. We found that this condition rarely worsened or developed in patients treated with ligation.

## CONCLUSION

In our population, endoscopic variceal ligation is a safe and effective technical approach. Moreover, the current study shows that using EBL as emergency endoscopic therapy added to vasoactive drugs is associated with a high rate of hemostasis. Variceal ligation is also effective and safe when beta-blockers are contraindicated or not tolerated for the prevention of first variceal bleeding. Concerning the secondary prophylaxis, combined therapy with propranolol seems to be more effective than endoscopic ligation for the prevention of recurrent bleeding.
